# Mechanisms of traditional Chinese medicine overcoming of radiotherapy resistance in breast cancer

**DOI:** 10.3389/fonc.2024.1388750

**Published:** 2024-06-27

**Authors:** Xiaohui Zhao, Ting Luo, Yuting Qiu, Zhiwei Yang, Danni Wang, Zairui Wang, Jiale Zeng, Zhuofei Bi

**Affiliations:** ^1^ Department of Oncology, Sun Yat-Sen Memorial Hospital, Sun Yat-Sen University, Guangzhou, China; ^2^ Department of Oncology, Shenshan Medical Centre, Memorial Hospital of Sun Yat-Sen University, Shanwei, China; ^3^ Guangdong Provincial Key Laboratory of Malignant Tumor Epigenetics and Gene Regulation, Sun Yat-sen Memorial Hospital, Sun Yat-sen University, Guangzhou, China; ^4^ Department of Radiology, Sun Yat-Sen Memorial Hospital, Sun Yat-sen University, Guangzhou, China

**Keywords:** breast cancer, radiation, radiotherapy resistance, herbal, apoptosis

## Abstract

Breast cancer stands as the most prevalent malignancy among women, with radiotherapy serving as a primary treatment modality. Despite radiotherapy, a subset of breast cancer patients experiences local recurrence, attributed to the intrinsic resistance of tumors to radiation. Therefore, there is a compelling need to explore novel approaches that can enhance cytotoxic effects through alternative mechanisms. Traditional Chinese Medicine (TCM) and its active constituents exhibit diverse pharmacological actions, including anti-tumor effects, offering extensive possibilities to identify effective components capable of overcoming radiotherapy resistance. This review delineates the mechanisms underlying radiotherapy resistance in breast cancer, along with potential candidate Chinese herbal medicines that may sensitize breast cancer cells to radiotherapy. The exploration of such herbal interventions holds promise for improving therapeutic outcomes in the context of breast cancer radiotherapy resistance.

## 1 Introduction

Breast cancer stands as the most prevalent cancer among women globally (1), ranking second in cancer-related mortality in female (2). Radiotherapy has become a widely employed therapeutic approach for breast cancer, especially in the context of breast-conserving surgery where it plays a crucial role in preventing local tumor recurrence post-surgery (3). Nevertheless, some patients experience local tumor recurrence after radiotherapy, possibly indicative of tumor resistance to radiation. Consequently, enhancing tumor sensitivity to radiation and overcoming radiotherapy resistance have emerged as pressing challenges in the field of breast cancer radiotherapy.

In recent years, multiple studies have reported the potential of traditional Chinese herbal medicine in increasing the sensitivity of breast cancer cells to radiotherapy, offering new avenues for exploring novel radiotherapy sensitization strategies. Traditional Chinese herbal medicine, with a history spanning thousands of years, encompasses various active constituents such as alkaloids, flavonoids, polysaccharides, tannins, and volatile oils, each imparting diverse pharmacological effects. With multiple therapeutic benefits, Chinese herbal medicine finds extensive application in traditional Chinese medicine for treating various acute and chronic conditions, including tumors.

Numerous researchers have dedicated efforts to unraveling the molecular mechanisms through which Chinese herbal medicine augments the sensitivity of breast cancer cells to radiotherapy. While the body of related research remains relatively limited, it has already yielded some promising advancements. Notably, Chinese herbal medicine not only exhibits the potential to enhance tumor sensitivity to radiation but may also mitigate radiation-induced damage to normal tissues to some extent, thereby improving the safety and efficacy of treatment.

As of now, there lacks a comprehensive and systematic review summarizing the existing research in this field. In this article, we conducted searches on databases such as PubMed, Scopus, and Web of Science, utilizing Medical Subject Headings (MeSH) terms and relevant free terms to enhance search sensitivity. Additionally, we reviewed key mechanisms associated with radiation resistance in tumor cells, summarized Chinese herbal medicines with radiosensitizing effects, and listed confirmed or potential herbal candidates that can enhance sensitivity to radiotherapy in breast cancer. This work aims to provide a targeted reference and guidance for future research in this promising domain.

## 2 Breast cancer overview

Breast cancer poses a significant threat to women’s health worldwide, standing as the most common malignancy and the second leading cause of cancer-related death among females (4). According to the ‘Cancer Statistics 2023’ report, breast cancer constitutes approximately 31% of female cancer cases, with an estimated addition of around 300,590 new diagnoses and over 43,700 deaths projected for 2023 (1). These statistics underscore the profound impact of breast cancer on women’s health. Globally, the incidence of breast cancer continues to rise, particularly in developed countries, driven by factors associated with the so-called ‘Western lifestyle,’ including poor dietary habits, smoking, excessive stress, and a lack of physical activity (5). Notably, in developing countries, despite a relatively lower incidence of breast cancer, the mortality rate surpasses that of developed nations.

Despite significant strides in early diagnosis and drug treatment for breast cancer in recent years, it remains a substantial challenge in the global landscape of women’s health. Exploring novel treatment strategies is therefore crucial for reducing breast cancer mortality rates.

Breast cancer is a highly heterogeneous and complex disease. Current clinical prognosis and treatment decisions are predominantly based on clinical characteristics and molecular subtypes. Clinical features encompass histological type, tumor size, lymph node involvement, and distant metastasis. Molecular subtypes are classified based on hormone receptor status, Her-2 expression, and the Ki67 index, resulting in luminal A, luminal B, HER-2 positive non-luminal, and triple-negative breast cancer (TNBC) (6). Luminal A, constituting 60% of breast cancers, is characterized by positive estrogen and/or progesterone receptors (ER+ and/or PR+), with HER2 negativity and a favorable prognosis (7). Luminal B subtype constitutes 30% of breast cancers and is characterized by high-risk factors within the Luminal category. It is further classified into HER2-negative or HER2-positive subtypes. Compared to Luminal A, Luminal B exhibits higher expression of proliferation-associated genes marked by Ki-67. It is associated with a poorer prognosis (8, 9). HER2-positive breast cancer accounts for 10%, displaying overexpression of HER2, while being ER- and PR-, and carrying an unfavorable prognosis (10). TNBC, constituting 15–20% of breast cancers, lacks expression of estrogen, progesterone, and HER2 receptors, contributing to lower treatment response and higher invasiveness, resulting in a poorer prognosis (11).

Beyond widely used molecular subtyping, recent advancements in tumor precision testing technologies, including genomics, transcriptomics, proteomics, metabolomics, and epigenomics, have gradually been applied in the diagnosis of breast cancer, providing personalized and precise treatment references for breast cancer patients (12–15). These technologies aid in genetic risk prediction, molecular subtype diagnosis, treatment efficacy prediction, and the selection of precision treatment strategies. For instance, next-generation sequencing is recommended for the detection of genetic susceptibility genes, high-frequency mutation genes, target drug-related genes, and resistance genes in breast cancer tissues and peripheral blood germ line DNA.

In 2018, the U.S. Food and Drug Administration (FDA) approved PARP inhibitors olaparib and talazoparib for the treatment of refractory metastatic breast cancer patients carrying harmful germline mutations in BRCA1/2. Additionally, the FDA approved the immune checkpoint inhibitor pembrolizumab for the treatment of advanced refractory breast cancer patients with mismatch repair deficiency or high microsatellite instability. Furthermore, for complex and refractory triple-negative breast cancer, several studies have utilized gene expression profiles to further classify it into different molecular subtypes, facilitating targeted therapy specific to each subtype. Currently recognized classification systems include Lehmann’s six types, Burstein’s four types, and the ‘Fudan classification’.

Regarding treatment, for non-metastatic breast cancer, the primary goal is complete eradication of the tumor in the breast and regional lymph nodes, aiming to prevent recurrence and metastasis. Local treatment primarily involves surgery, with postoperative adjuvant radiotherapy required for high-risk and breast-conserving surgery patients. Systemic treatment for non-metastatic breast cancer can be administered preoperatively (neoadjuvant therapy) or postoperatively (adjuvant therapy). Treatment plans are tailored based on specific molecular subtypes, such as hormone receptor-positive patients requiring endocrine therapy (some patients also receiving chemotherapy), HER2-positive patients receiving HER2-targeted antibody therapy, often combined with chemotherapy, and triple-negative breast cancer primarily treated with chemotherapy. For metastatic breast cancer, the main treatment goal is to prolong patient survival and improve symptoms. Systemic treatment takes precedence, with surgery and radiotherapy primarily used to alleviate symptoms (16).

## 3 Mechanisms underlying tumor radioresistance

The fundamental principle of radiotherapy is to utilize radiation or ionizing energy to damage molecular structures, aiming to achieve the goal of killing tumor cells. Radiotherapy primarily kills tumor cells through the following mechanisms: direct effects and indirect effects (17). Direct effects involve the direct action of radiation or ionizing energy on molecular structures within cells, especially DNA, leading to DNA damage, including double-strand breaks (DSB), single-strand breaks (SSB), and damage to nucleotide bases. Indirect effects result from the release of radiation energy, causing water molecules in the intracellular and extracellular environments to undergo radiation attacks, generating highly reactive and unstable reactive oxygen species (ROS) such as superoxide radicals (O2-) and hydroxyl radicals (OH). These ROS further trigger oxidative stress (OS) within the cells, causing cellular damage.

In addition to direct and indirect effects, radiotherapy can also influence neighboring cells that are not directly irradiated through the bystander effect. This implies that irradiated cells can release signals, transmitting apoptosis signals to surrounding non-irradiated cells through direct cell contact or intercellular communication, inducing similar biological effects as the irradiated cells. Overall, these effects collaborate to cause DNA damage, chromosomal instability, mutations, and apoptosis in cancer cells, ultimately leading to their demise. These mechanisms constitute the basis of how radiotherapy kills tumor cells.

Theoretically, radiotherapy should be effective against all tumor cells; however, in reality, there are certain tumors that exhibit poor responsiveness to radiotherapy, leading to a higher risk of recurrence and metastasis. This is primarily attributed to the radioresistance of tumor cells. The radioresistance of tumors is a complex issue, involving various mechanisms, including the heterogeneity of tumor cells and the influence of the tumor microenvironment. In-depth exploration of the specific mechanisms underlying tumor radioresistance is crucial for developing strategies to enhance the efficacy of radiotherapy. Current research has summarized the major mechanisms influencing tumor sensitivity to radiotherapy, encompassing the following aspects: DNA damage repair, cell cycle arrest, tumor stem cells, alterations in the tumor microenvironment, extracellular vesicles and non-coding RNA, metabolic reprogramming, and ferroptosis. We have summarized the mechanisms underlying radiation resistance in breast cancer in **Table 1**.

**Table 1:** Mechanisms underlying radiotherapy resistance in breast cancer.

Substance	Mechanism	References
DNA damage repair	DNA damage repair mechanisms include mismatch repair, base excision repair, nucleotide excision repair, and double-strand break repair, among others. When DNA damage occurs, DNA damage checkpoints are activated first. DNA damage sensors such as ATRIP, Rad24p, γH2AX, NBS1, BRCA1/2, Ku70/80, and RNA polymerase recognize the damage signals and recruit core kinases of the DNA damage response (DDR). These kinases then recruit and activate enzymes involved in the DNA damage repair process, such as PNKP, Tdp-1, and APE-1.	(18–28)
Cell cycle arrest	Ionizing radiation activates the ATM (Ataxia-telangiectasia mutated) protein, a process that involves dimer dissociation, autophosphorylation, and phosphorylation of downstream proteins such as p53 and Chk2. High expression of ATM is associated with radiation resistance. Additionally, tumor cells exposed to radiation induce breaks in their own DNA by activating the expression of Caspase-activated DNase (CAD), leading to arrest in the G2 phase of the cell cycle and subsequent DNA damage repair.	(29–33)
Apoptosis	Following irradiation, radiation-resistant tumor cells can inhibit apoptosis by modulating the interaction network of the Bcl family of proteins. This modulation involves upregulation of anti-apoptotic proteins such as Bcl-2 and Bcl-XL, as well as downregulation or inactivation of pro-apoptotic proteins such as Bax and Bak.	(34–41)
Regulation of Cancer Stem Cells	Tumor stem cells enhance their resistance to radiation therapy through various mechanisms, including dormancy, enhanced DNA repair capacity, upregulation of cell cycle control mechanisms, scavenging of free radicals, and interactions with the stromal components in the tumor microenvironment. These adaptations collectively contribute to the increased resistance of tumor cells to radiotherapy.	(42–51)
Ferroptosis	Overexpression of the iron death suppression genes GLC7A11 and GPXA can induce resistance to tumor radiotherapy.	(52–54)
Regulation of Tumor microenvironment	Hypoxic conditions can induce the transition of tumor cells from an epithelial phenotype to a mesenchymal phenotype and enhance radiation resistance through increased levels of hypoxia-inducible factor 1 (HIF-1). Cancer-associated fibroblasts (CAFs) induce epithelial-mesenchymal transition in tumor cells and secrete CXCL1, which inhibits the expression of the reactive oxygen species (ROS) scavenging enzyme superoxide dismutase 1. This leads to increased ROS accumulation after radiation, resulting in radiation resistance.	(55–68)

### 3.1 DNA damage repair

As previously discussed, ionizing radiation induces a broad spectrum of DNA damage through direct effects, indirect effects, and bystander effects. These damages primarily include double-strand breaks (DSBs), single-strand breaks, base damage, and interstrand cross-links, with DSBs being the most severe form of damage (18). These DNA lesions can pose significant obstacles to the adaptation and survival of tumor cells, thereby promoting cell death. However, tumor cells gradually evolve a series of complex and intricate mechanisms to cope with these damages, primarily through the activation of DNA damage repair pathways. DNA damage repair mechanisms encompass mismatch repair, base excision repair, nucleotide excision repair, and double-strand break repair.

**Figure 1:**
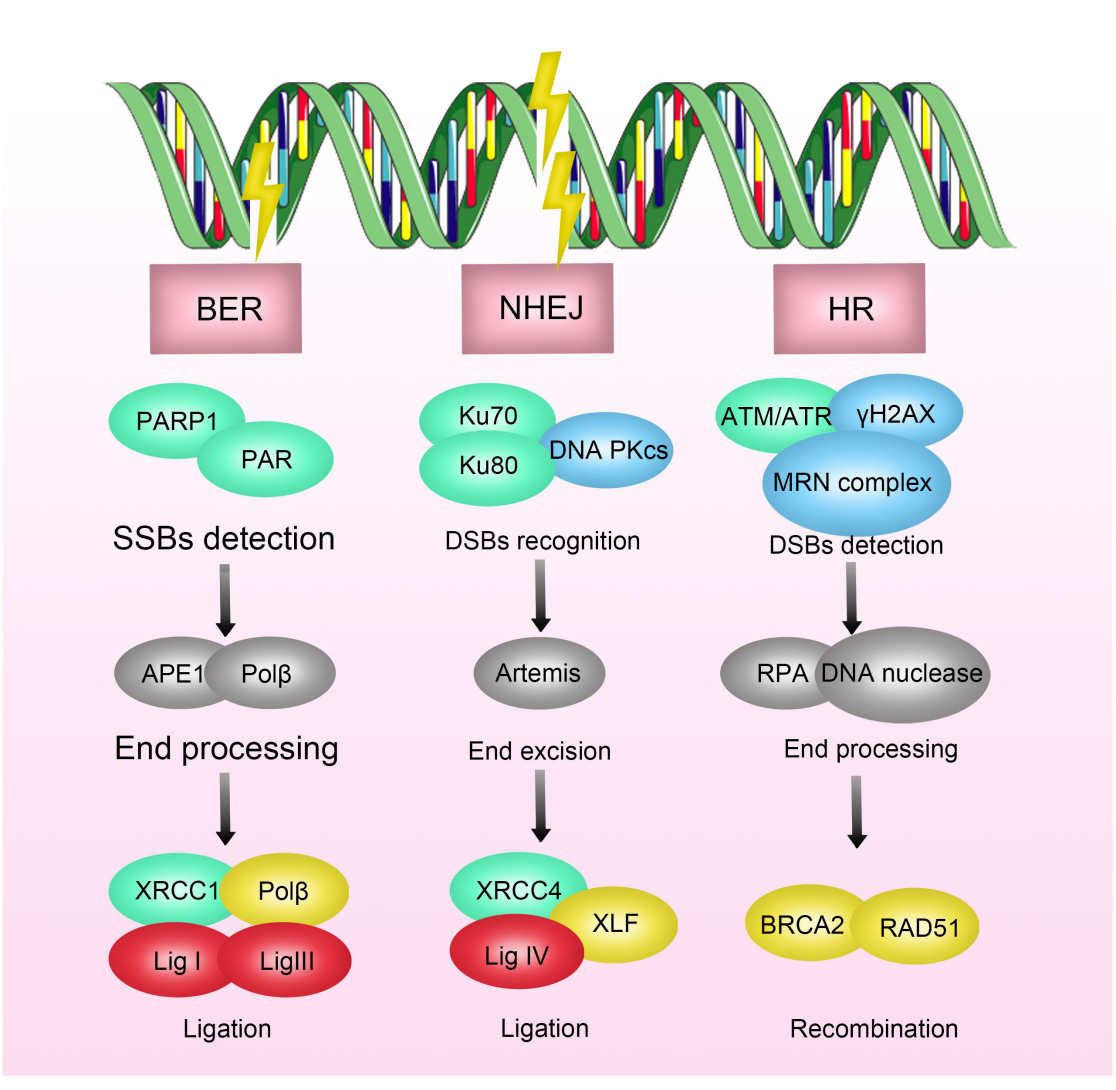
Mechanisms of Radioresistance Caused by DNA Damage Repair in Breast Cancer. DNA damage includes double-strand breaks, single-strand breaks, base damage, and interstrand cross-links. Radiation-induced DNA damage response activates the DNA repair pathway, where double-strand breaks (DSBs) are primarily repaired through non-homologous end joining (NHEJ) and homologous recombination (HR). DNA damage triggers DNA damage sensors, which recognize the damage signals and recruit core kinases of DNA damage response (DDR), thereby activating enzymes involved in DNA damage repair processes. Inhibition of key enzymes in DNA damage repair has been shown to enhance the efficacy of radiotherapy.

In the process of DSB repair, non-homologous end joining (NHEJ) and homologous recombination are two critical pathways. When DNA damage occurs, the DNA damage checkpoint is initially activated, delaying mitosis, and providing more time for DNA repair (19–21). Subsequently, DNA damage sensors such as ATRIP, Rad24p, γH2AX, NBS1, BRCA1/2, Ku70/80, and RNA polymerase recognize damage signals and recruit core kinases of DNA damage response (DDR), including Ataxia Telangiectasia Mutated protein kinase (ATM), Ataxia Telangiectasia and Rad3-related protein kinase (ATR), and DNA-dependent protein kinase (DNA-PK) (22–26). Upon activation, these kinases recruit and activate enzymes involved in the DNA damage repair process, such as PNKP, Tdp-1, and APE-1. Ultimately, the XPCC4-XLF-LIG4 complex reconnects the broken ends of DNA (27, 28) (**Figure 1**).

Inhibitors targeting key enzymes in the DNA damage process have been shown to enhance the efficacy of radiotherapy. For example, DNA-PKcs inhibitors have been demonstrated to increase the sensitivity of multiple myeloma, nasopharyngeal carcinoma, and glioblastoma to radiotherapy (69–71). Similarly, inhibitors of the DNA repair enzyme poly (ADP-ribose) polymerase (PARP) have been shown to increase the sensitivity of breast cancer, prostate cancer, pancreatic cancer, and ovarian cancer to radiotherapy (72–74). In breast cancer, preliminary clinical evidence supports this notion. A clinical trial focusing on triple-negative breast cancer suggests that the combined application of radiotherapy and PARP inhibitors, especially in the presence of BRCA1/2 mutations, can enhance the efficacy of radiotherapy by indirectly increasing the frequency of unrepaired DSBs in the base excision repair pathway, demonstrating good safety and tolerability (75).

Furthermore, inhibitors targeting single-stranded DNA binding protein Replication protein A (RPA) and X-ray repair cross-complementing 1 (XRCC1) have also shown the potential to increase the sensitivity of various tumors, including breast cancer, liver cancer, and head and neck squamous cell carcinoma, to radiotherapy (76).

### 3.2 Cell cycle arrest

The cell cycle is a collective term for a series of events in the cellular life process that promote cell growth and division into two new daughter cells. The cell cycle primarily consists of four stages: G1 phase (the growth phase before DNA synthesis), S phase (DNA replication/synthesis phase), G2 phase (the final preparation phase before cell division), and M phase (mitosis) (77, 78). The regulation of the cell cycle depends on a series of checkpoints that ensure cells must correctly complete all crucial processes of the current stage before entering the next stage. For instance, the G1/S checkpoint ensures the cell’s size and DNA integrity are suitable for DNA replication, while the G2/M checkpoint ensures the accuracy of the DNA replication process and prepares the cell for division (79).

In normal cells, the P53 protein plays a crucial regulatory role in the G1/S checkpoint. When DNA damage occurs, the action of the P53 protein leads to a G1 phase pause, preventing entry into the S phase and initiating DNA damage repair mechanisms. Conversely, in tumor cells, the regulatory factors of the G1/S checkpoint often malfunction, allowing tumor cells with DNA damage to easily progress into the S phase (80). However, tumor cells can buy time for DNA damage repair by inducing arrest in the G2/M phase (81).

**Figure 2:**
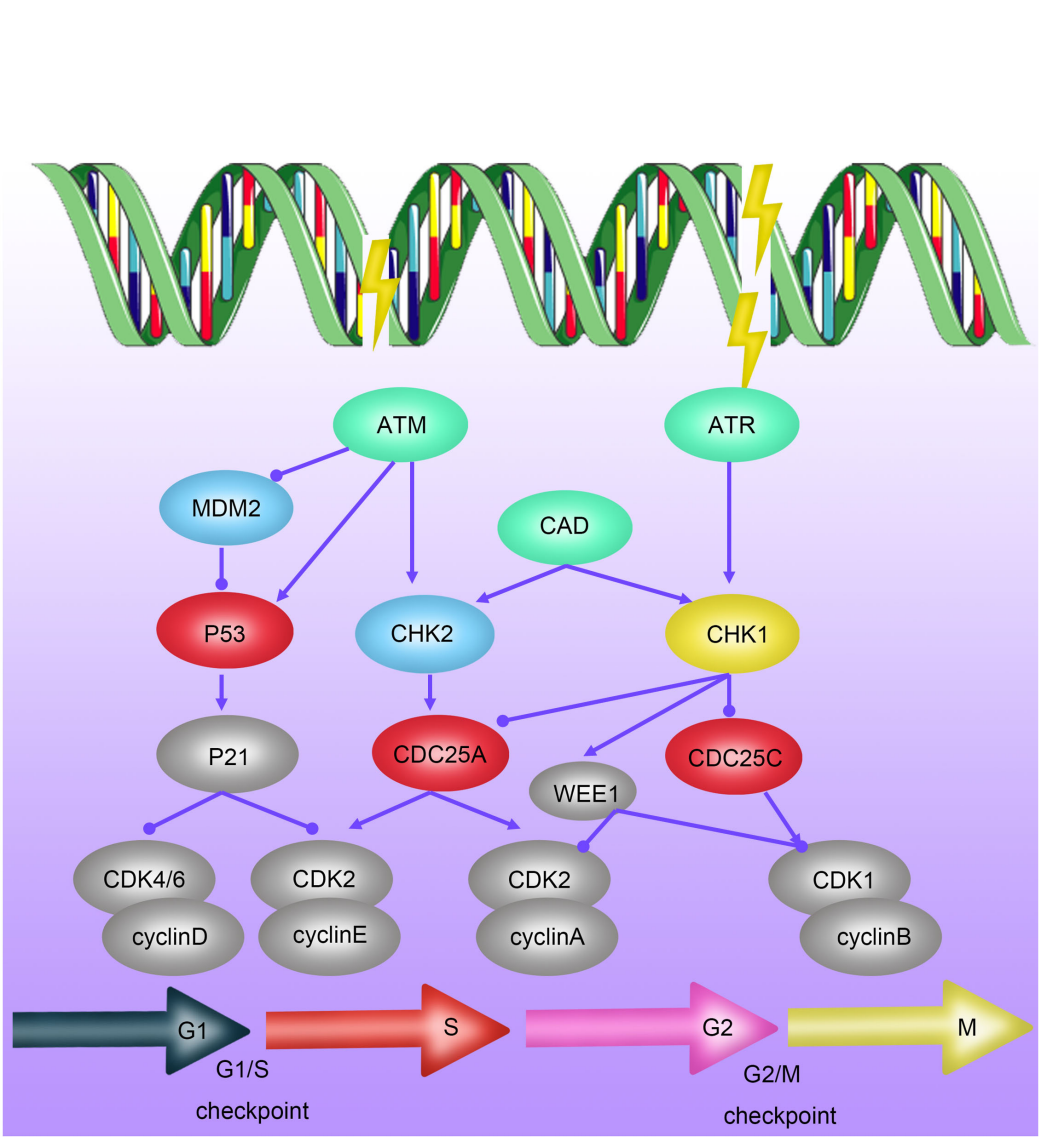
Mechanisms of Radioresistance Caused by Cell Cycle Arrest in Breast Cancer. Cell-cycle checkpoints are activated in response to DNA damage induced by ionizing radiation (IR). ATM kinase is primarily activated by DNA double-strand breaks (DSBs), mediating the initial response to DSBs and cell-cycle arrest through CHK2 activation. P53 activates the G1/S checkpoint via p21, promoting DNA repair or inducing apoptosis or senescence. DNA single-strand breaks (SSBs) activate ATR kinase, which, through CHK1 action, further activates the S-phase checkpoint and G2/M checkpoint.

ATM (Ataxia-telangiectasia mutated) protein is a critical regulator of cell cycle checkpoints and belongs to the phosphatidylinositol 3-kinase-related protein kinase family. Under the influence of ionizing radiation, the ATM protein becomes activated, a process involving dimer dissociation, autophosphorylation, and phosphorylation of downstream proteins (such as p53 and Chk2). As a key regulator of the cell cycle checkpoint, the P53 protein induces G1/S phase arrest by activating p21 protein. Simultaneously, the Chk2 pathway can regulate the transition of the G2/M phase when the cell division cycle protein 2 (Cdc2)/cyclinB complex is activated (29–32) (**Figure 2**). Studies have shown that elevated expression of ATM is associated with radioresistance in various tumors, including breast cancer, lung cancer, and gliomas, while ATM inhibitors can enhance the sensitivity of these tumor cells to radiotherapy (33).

Recent research has also revealed a novel tumor G2/M phase arrest mechanism: tumor cells exposed to radiation induce the cleavage of their own DNA by activating the expression of Caspase-activated DNase (CAD), thereby achieving arrest in the G2 phase of interphase cell division and gaining time for DNA damage repair caused by radiotherapy (29). In summary, G2/M phase arrest plays a crucial role in the radioresistance of tumor cells, and targeting cell cycle arrest at this stage becomes a significant focus for improving the sensitivity of tumor radiotherapy.

### 3.3 Apoptosis

**Figure 3:**
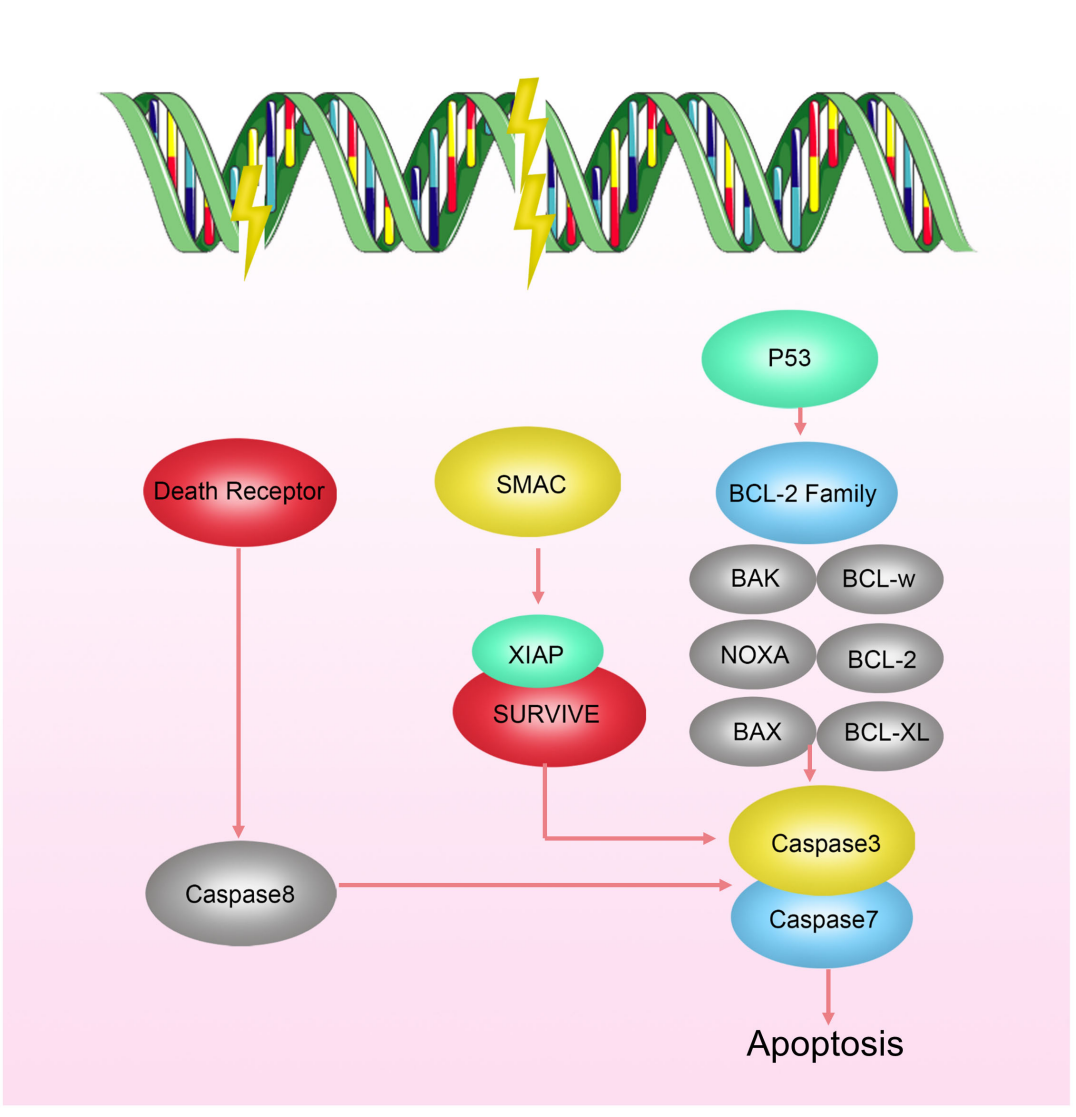
Mechanisms of Radioresistance Related to Apoptosis in Breast Cancer. Cell apoptosis occurs via intrinsic pathways regulated by the Bcl-2 family activating CaspaseC or extrinsic pathways mediated by the expression of death receptor ligands. Radiation-resistant cells can inhibit apoptosis by modulating the interaction network of the Bcl family.

Apoptosis, also known as programmed cell death, is a finely regulated process of cellular self-destruction. It can occur through intrinsic pathways (involving Bcl-2-mediated mitochondrial cytochrome c release) or extrinsic pathways mediated by the expression of death receptor ligands (34). Apoptosis plays a crucial role in various biological processes, including organism development, tissue remodeling, and responses to cellular damage or diseases. Under normal physiological conditions, apoptosis contributes to maintaining stable cell numbers and eliminating damaged or abnormal cells, preventing them from becoming potential sources of pathology. Apoptosis is closely related to the occurrence and treatment of tumors. “Apoptosis evasion” is an important survival capability of tumor cells to protect themselves from radiation damage when DNA damage repair fails (35). Research has shown that radiation-resistant cells can inhibit apoptosis by modulating the Bcl family interaction network (**Figure 3**). Upregulating anti-apoptotic proteins such as Bcl-2 and Bcl-XL, and downregulating or inactivating pro-apoptotic proteins such as Bax and Bak, has been confirmed to increase radiation resistance of malignant tumors, including breast cancer, lung cancer, mesothelioma, pancreatic cancer, to radiotherapy (36–41).

### 3.4 Regulation of cancer stem cells

**Figure 4:**
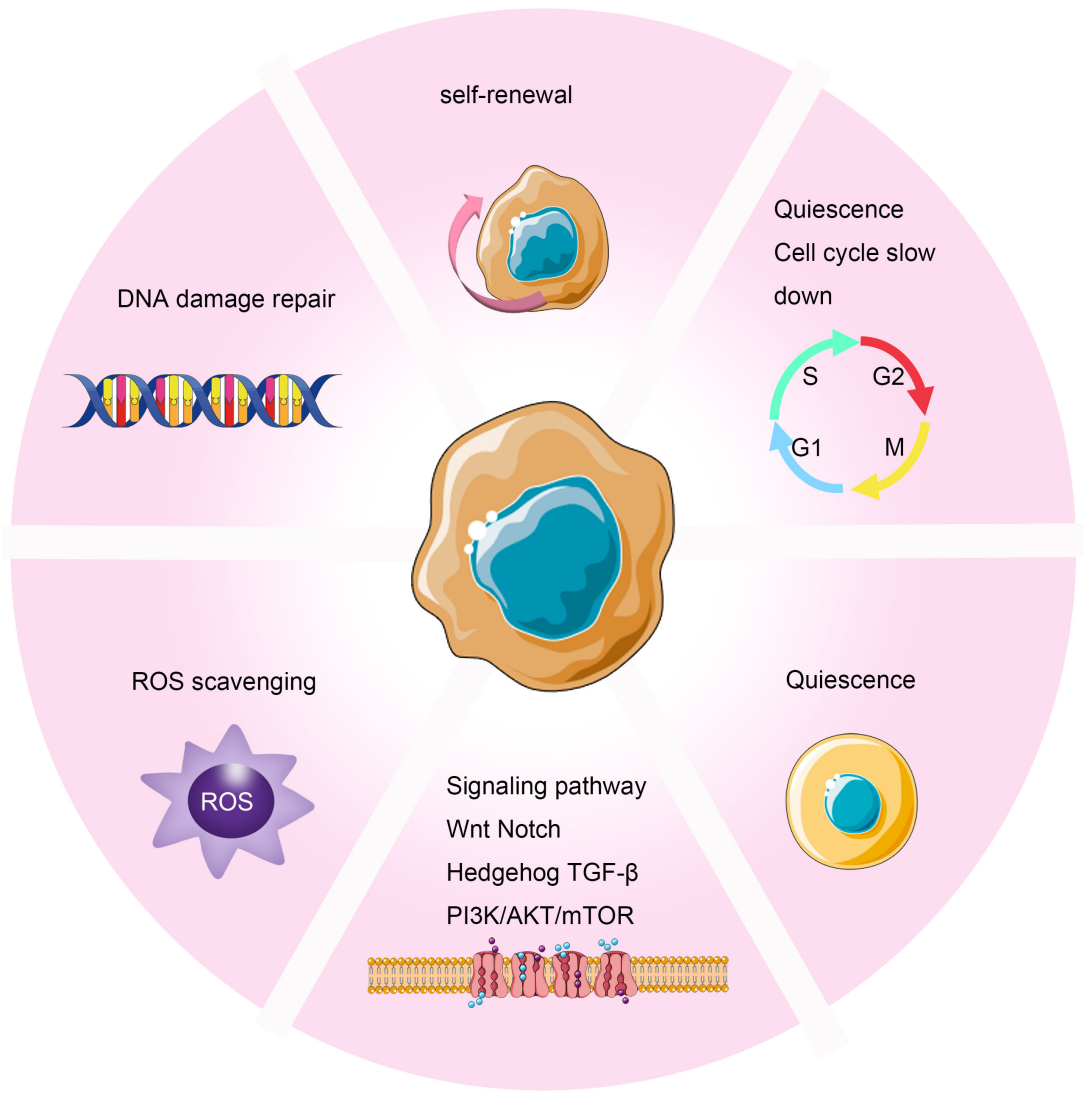
Mechanisms of Radioresistance Caused by Cancer Stem Cells in Breast Cancer. Cancer stem cells induce radioresistance through enhanced DNA repair capability, self-renewal, cell cycle arrest, scavenging of free radicals, dormancy, and interactions with stromal components in the tumor microenvironment.

In the field of radiation oncology, the exploration of Cancer Stem Cells (CSCs) has become a crucial research direction. As a unique cellular subset within tumors, CSCs possess the characteristics of sustained self-renewal and differentiation, widely recognized as the major driving force behind tumor recurrence and metastasis (82). Despite their relatively small proportion within the overall tumor cell population, they exhibit potent tumorigenic capabilities and can survive in adverse growth environments, propelling tumor development and spread (83, 84). Current research indicates that the mechanisms through which CSCs develop resistance to radiotherapy involve multiple aspects (42–46). These include the dormant tendency of CSCs, enabling them to evade attacks during radiotherapy; enhanced DNA repair capabilities, allowing effective repair of radiation-induced DNA damage; upregulated cell cycle control mechanisms, further reinforcing their resistance to treatment; and robust free radical clearance abilities, helping reduce oxidative stress caused by radiotherapy (47). Additionally, CSCs interact specifically with stromal components in the tumor microenvironment, enhancing their survival and proliferative capacities (48–51) (**Figure 4**). To overcome this radiotherapy resistance, researchers have begun exploring inhibitors targeting specific signaling pathways in CSCs, such as inhibitors against Notch and Wnt/β-catenin. Early clinical trials of these novel drugs have shown potential in improving tumor sensitivity to radiotherapy. Through this approach, combining traditional radiotherapy with targeted treatments against CSCs could pave the way for new avenues to enhance the effectiveness of cancer therapy.

### 3.5 Ferroptosis

**Figure 5:**
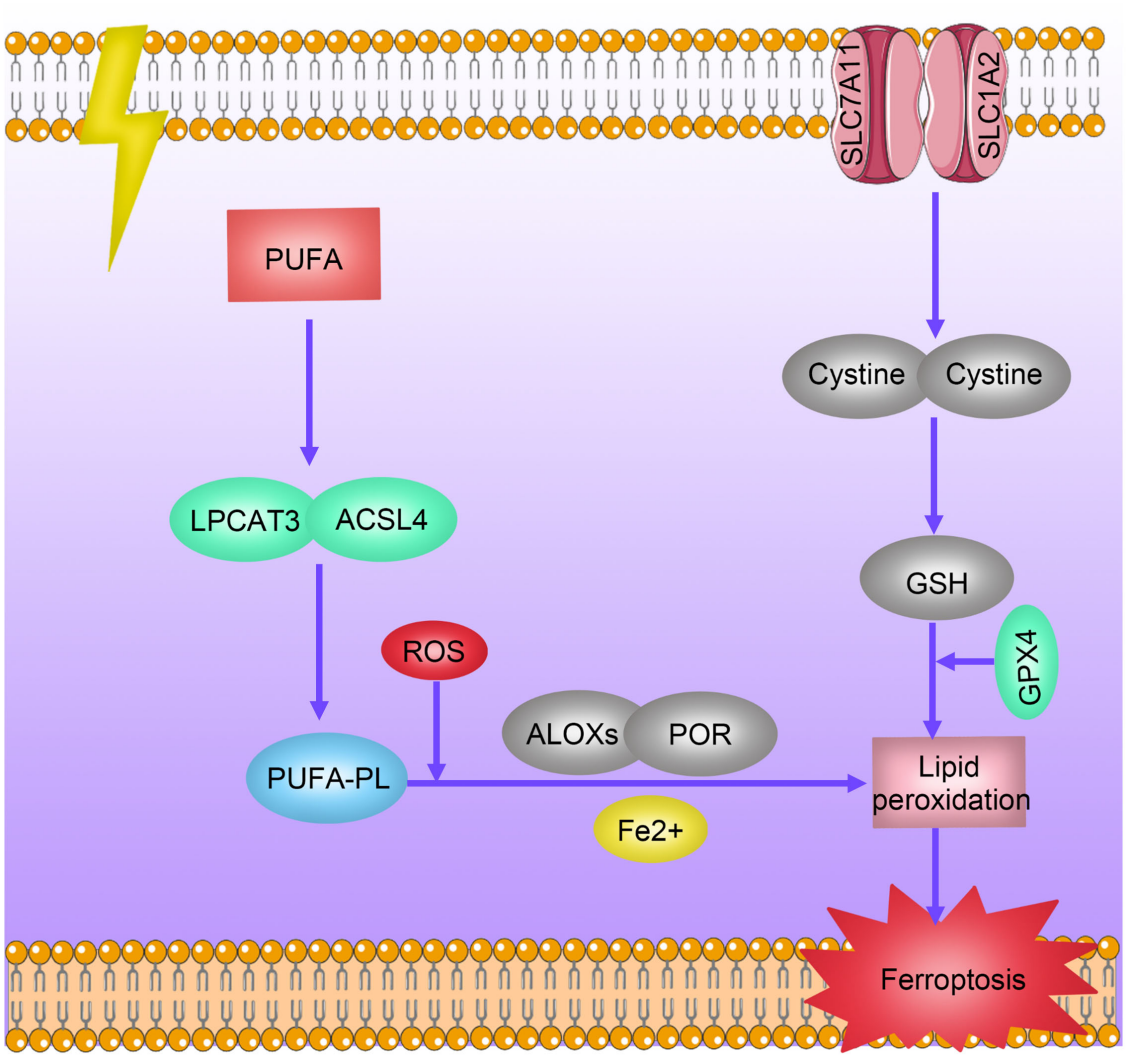
Mechanisms of Radioresistance Caused by Ferroptosis in Breast Cancer. Radiotherapy induces ferroptosis in tumor cells through multiple pathways. Radiation promotes lipid peroxidation and ferroptosis by inducing reactive oxygen species (ROS) production and overexpression of ACSL4 and LPCAT3. Radiation decreases the expression levels of antioxidant system subunits SLC7A11 and SLC1A2 by activating the p53 pathway, thereby reducing their inhibition of glutathione synthesis levels and promoting ferroptosis. Overexpression of ferroptosis-inhibiting genes SLC7A11 and GPXA induces tumor radiotherapy resistance.

Ferroptosis is a specific type of cell death induced by lipid peroxidation and relies on the presence of iron ions. Unlike traditional forms of cell death such as apoptosis, necrosis, or autophagy, ferroptosis is initiated by lipid peroxidation leading to membrane damage, ultimately triggering cell death (52). Research indicates that radiotherapy can induce iron death in tumor cells through various mechanisms (53, 54). On one hand, radiotherapy can promote lipid peroxidation and iron death by inducing the generation of reactive oxygen species (ROS) and overexpression of ACSL4, a lipid metabolism enzyme essential for iron death. On the other hand, radiotherapy can reduce the expression of the antioxidant system subunit SLC7A11 through activation of the p53 pathway, thereby alleviating its inhibitory effect on glutathione synthesis and promoting iron death. Conversely, overexpression of iron death inhibitory genes, such as SLC7A11 and GPXA, can induce resistance to radiotherapy in tumors (53) (**Figure 5**).

### 3.6 Regulation of tumor microenvironment

**Figure 6:**
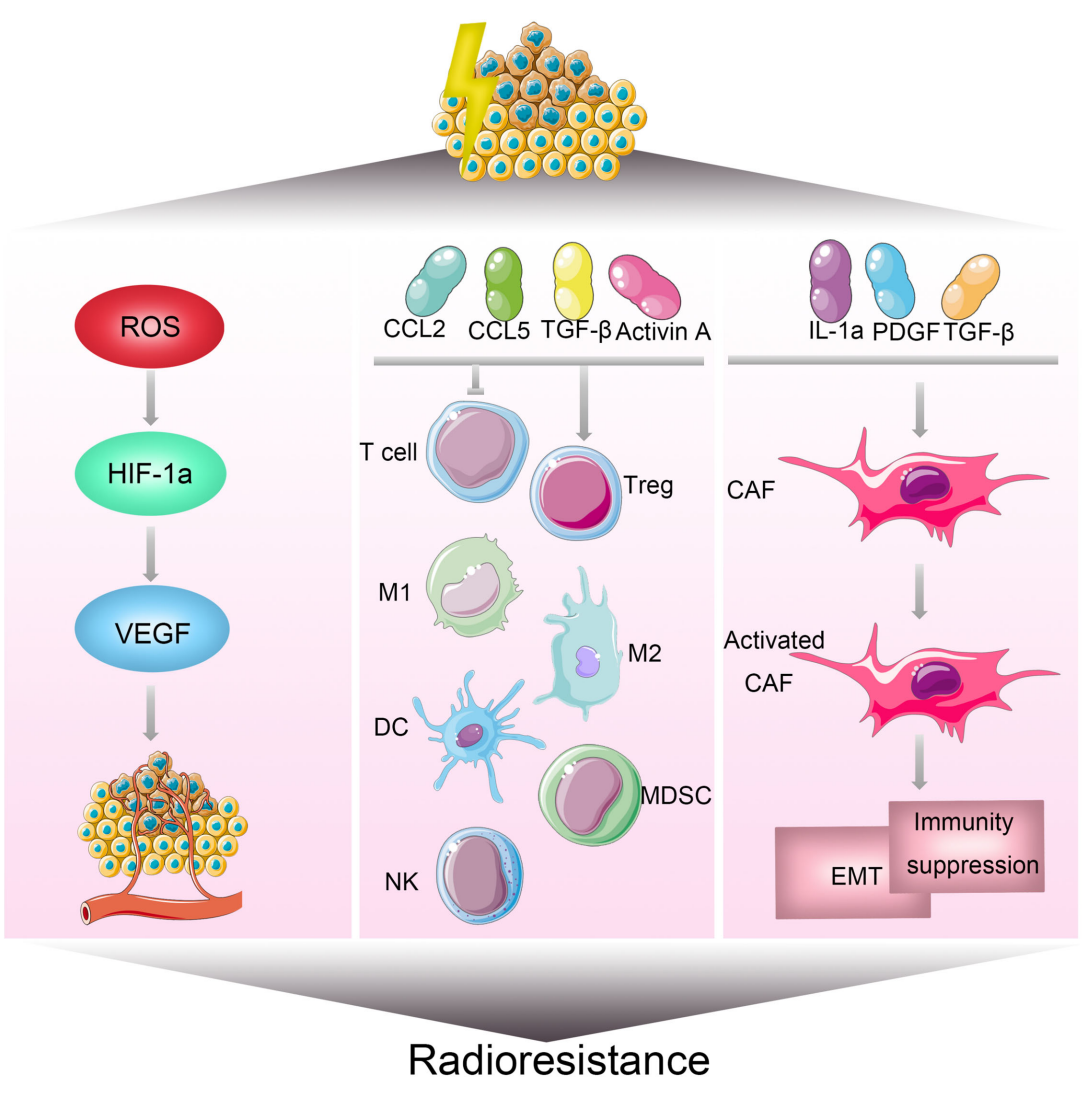
Mechanisms of Radioresistance Associated with the Tumor Microenvironment in Breast Cancer. The tumor microenvironment is a significant factor influencing the sensitivity of breast cancer to radiotherapy. Hypoxia is one of the key characteristics within the tumor microenvironment. On one hand, hypoxia can induce the transition of tumor cells from an epithelial phenotype to a mesenchymal phenotype. On the other hand, it can increase the expression of hypoxia-inducible factor 1 (HIF-1), promoting the secretion of vascular endothelial growth factor (VEGF) by tumor cells, thereby enhancing radiation resistance. The tumor microenvironment comprises extracellular matrix, various chemokines and cytokines, vascular networks, immune cells, and numerous other molecules. Alterations in the tumor microenvironment contribute to radiotherapy resistance, leading to cell survival, angiogenesis, and tumor growth in breast cancer.

Tumor microenvironment is a critical area in the growth and development of tumors, characterized by its complexity in features and components. This environment not only encompasses tumor cells but also includes extracellular matrix, various chemokines and cytokines, vascular networks, immune cells, and numerous other molecules (85, 86). Studies indicate that hypoxia, a low-oxygen condition, is one of the important features of the tumor microenvironment (55). Under hypoxic conditions, tumor cells can exhibit 2 to 3 times enhanced resistance to radiation (56). Hypoxia contributes to increased resistance to radiation in several ways. On one hand, it induces tumor cells to undergo epithelial-to-mesenchymal transition, enhancing resistance to radiation therapy (57). On the other hand, hypoxia leads to the upregulation of hypoxia-inducible factor-1 (HIF-1). HIF-1 promotes the secretion of VEGF by tumor cells, protecting tumor vascular endothelial cells and increasing their tolerance to radiation (58) (**Figure 6**). Moreover, HIF-1 activation can stimulate key glycolytic enzymes to generate NADPH and glutathione, clearing reactive oxygen species (ROS) produced after radiation and reducing DNA damage (59, 60). Additionally, the production of a large amount of lactate is believed to enhance tumor radioresistance through the GPR81/mTOR/HIF-1/STAT3 pathway (61). Preliminary clinical studies suggest that hyperbaric oxygen therapy and hypoxia-activated prodrugs, such as nitroimidazole, may improve the effectiveness of radiotherapy (62). Cancer-associated fibroblasts (CAFs) in the tumor microenvironment also play a crucial role in influencing tumor radiotherapy sensitivity (**Figure 6**). These cells can secrete various cytokines, growth factors, chemokines, and extracellular matrix remodeling molecules, promoting tumor growth (63–65) Studies show that CAFs, on one hand, induce epithelial-mesenchymal transition in tumor cells, enhancing their resistance to radiotherapy (66, 67). On the other hand, CAF-secreted CXCL1 inhibits the expression of the ROS scavenging enzyme superoxide dismutase 1, leading to increased ROS accumulation after radiation, thereby strengthening DNA damage repair and mediating radiation resistance (68). Furthermore, the tumor immune microenvironment is an indispensable part of the tumor microenvironment and plays a critical role in the development of radiation resistance in tumors (87)(**Figure 6**). For instance, Ionizing radiation can upregulate PD-L1 expression through various pathways, diminishing the cytotoxic effects of CD8+ cytotoxic T lymphocytes (CTLs) against tumors. Combining radiotherapy with anti-PD-L1 therapy has been found to reduce immune escape and enhance the anti-tumor effects of radiotherapy. Furthermore, the combination of radiotherapy with anti-CTLA-4 and other immune modulatory therapies can synergize the effect of radiotherapy. Additionally, radiotherapy promotes the release of immune-suppressive chemokines CCL2 and CCL5, activates the immune-suppressive cytokine TGF-β, secretes activin A, and locally accumulates extracellular adenosine, collectively resulting in the recruitment of regulatory T cells, immunosuppressive (M2-type) macrophages, and myeloid-derived suppressor cells (MDSCs), impeding the activation and function of CD8+ T cells, mediating tumor immune resistance, and inhibiting the efficacy of radiotherapy (88, 89).

## 4 Traditional Chinese medicine and radiotherapy resistance

Traditional Chinese Medicine (TCM) has a history spanning thousands of years in the treatment of tumors and is widely applied in the comprehensive therapy of cancer. In addition to being used independently, TCM is often employed as an adjunctive measure, combined with conventional treatments such as surgery, radiotherapy, and chemotherapy, to enhance the overall effectiveness of cancer treatment. Chinese herbal medicine is believed to offer substantial advantages, including the inhibition of tumor progression, improvement of the efficacy of chemotherapy and radiotherapy, enhancement of immune system function, and reduction of side effects. Unlike Western medicines composed of purified compounds, traditional Chinese herbal formulations may consist of multiple herbs and components, acting on various cellular mechanisms and molecular targets simultaneously. Even compounds isolated from herbs may exhibit multiple effects. Traditional Chinese Medicine has shown promise in the treatment of breast cancer. It has been demonstrated to have beneficial effects in conjunction with radiotherapy by altering molecular signaling pathways, making it an effective agent for enhancing radiosensitivity (**Table 2**).

**Table 2:** Mechanisms of radiotherapy sensitivity by combined traditional Chinese medicine and radiotherapy.

Substance	Mechanism	References
Curcumin	MiRNA Modulators, Downregulation of miR-21/PTEN/Akt Pathway, Promotion of Apoptosis, Induction of Apoptosis, Regulation of Cell Cycle, and Elevation of ROS Levels.	(90–94)
Scutellaria baicalensis	NF-κB, Wnt/β-catenin, SATB1, Bcl2 family proteins, Caspase, PI3K/Akt, mTOR, ERK, p38-MAPK, TGF-β/Smad, and Hippo/YAP pathways increase the sub-G1 phase ratio, suppress mitochondrial membrane potential, induce apoptosis by downregulating Bcl-2 and upregulating Bax, caspase-dependent apoptosis, block S and G2/M phases, upregulate IFIT2 to inhibit tumor stem cells, and inhibit the Nrf2/HIF-1α pathway.	(95–103)
Artemisinin	Increase micronucleus frequency (MNF) and micronucleated cell frequency (MNCF), inhibit stem cell phenotype, β-catenin, and MMP-9, trigger reactive oxygen species (ROS) production and inhibit glutathione-S-transferase (GST) activity, increase apoptosis induction, cell cycle arrest, and impede DNA damage response, and increase NO generation inducing cell cycle arrest at the G2/M phase.	(104–109)
Resveratrol	Autophagy, regenerative gene (REG) III expression, hinders DNA damage repair, G2/M arrest of the cell cycle, induces necrosis and aging, decreases Bax/Bcl-2 ratio and increases caspase 8 activity, affects oxidative cell metabolism, enhances the expression of apoptotic genes Bax, p53, and caspase 8, inhibits HIF-1α, inhibits angiogenesis, and induces anti-tumor immunity.	(110–118)
Huaier	Autophagy, expression of Regenerating islet-derived protein III (REGIII), hinder DNA damage repair, cell cycle G2/M phase arrest, induce necrosis and senescence, decrease Bax/Bcl-2 ratio and enhance caspase 8 activity, impact oxidative cellular metabolism, enhance expression of apoptosis genes Bax, p53, caspase 8, inhibit HIF-1α, suppress angiogenesis, induce anti-tumor immunity.	(119–122)
Berberine	Inhibit cell cycle progression, promote cell apoptosis, suppress tumor stem cells, induce G2/M phase cell cycle delay, downregulate homologous recombination repair protein RAD51, induce mitochondrial ROS production and activate mitochondrial apoptosis, regulate tumor microenvironment via PI3K/HIF-1 pathway to overcome hypoglycemia and hypoxia, target Rad51 and epithelial-mesenchymal transition.	(123–135)
Triptolide and celastrol	Reduce HMGB1 expression, induce autophagy and affect phosphorylation of p38 mitogen-activated protein kinase, extracellular signal-regulated kinase (Erk) 1/2, and mammalian target of rapamycin (mTOR), inhibit GRP78 protein expression, induce cell apoptosis, M/G2 phase arrest, suppress the PI3K/Akt signaling pathway, increase PARP cleavage, JNK and p53 expression, inhibit HSP70 and Akt expression, modification by the antioxidant thiol molecule.	(136–144)
Withaferin	Increase ROS production levels, upregulate PARP cleavage, downregulate Bcl-2 expression, and activate JNK and p38 signaling pathways, leading to G2/M and pre-G1 phase arrest, as well as modulation of BCL2/Bax signaling cascade.	(145–147)
CAPE	Induce γH2AX foci and cell apoptosis, enhance Akt/mTOR phosphorylation, hinder cell migration, reduce the expression of DNA repair proteins RAD50 and RAD51, induce cell cycle arrest at S/G2 phase, elevate reactive oxygen species (ROS) levels, modulate lncRNA, deplete GSH, and inhibit NF-κB activity.	(148–153)

### 4.1 Curcumin

Curcumin, derived from Curcuma longa, is a diarylheptanoid also known as diferuloylmethane [1,7-bis(4-hydroxy-3-methoxyphenyl)-1,6-heptadiene-3,5-dione]. It is a natural antioxidant with various bioactivities believed to be beneficial for human health. Curcumin’s main actions include antioxidation, anti-inflammation, anti-tumor, immune modulation, and neuroprotection. The potential anti-tumor properties of curcumin are mediated through multiple mechanisms, including the inhibition of cell proliferation, migration, invasion, angiogenesis, induction of G2/M cell cycle arrest, apoptosis, and paraptosis.

Studies indicate that curcumin appears to be a crucial miRNA regulator in breast cancer. Curcumin interacts with various carcinogenic and anti-cancerous miRNAs involved in different stages of breast cancer. After treating various breast cancer cell lines with curcumin, miR181b, miR-34a, miR-16, miR-15a, and miR-146b-5p are upregulated, while miR-19a and miR-19b are downregulated. These effects lead to the inhibition of tumor occurrence and metastasis, along with the induction of apoptosis (90). In breast cancer, Wang et al. found that curcumin inhibits the proliferation and promotes apoptosis of MCF-7 breast cancer cells. The mechanism is associated with curcumin downregulating the miR21/PTEN/Akt pathway expression (91).

In breast cancer, based on transcriptomics and metabolomics research, Minafra et al. discovered that curcumin, in combination with radiotherapy, induces molecular imbalances involved in apoptosis induction, inflammatory processes, cell cycle regulation, and tyrosine metabolism in MCF7 and MDA-MB-231 breast cancer cells. Specifically, curcumin, when used in combination with radiation therapy, has been demonstrated to have dual effects on promoting cell apoptosis. Firstly, it can increase the levels of intracellular calcium ions (Ca2+) and reactive oxygen species (ROS) within the mitochondria, thereby enhancing mitochondrial permeability and facilitating the formation of apoptotic bodies, thus participating in intrinsic apoptosis. Secondly, curcumin can also upregulate the expression levels of “death activators” such as Fas ligand, TRAIL, and TNF-α, thereby participating in extrinsic apoptosis. On the other hand, curcumin sensitizes cells to radiation therapy by inhibiting the expression of various proteins associated with cell survival, proliferation, angiogenesis, invasion, and metastasis, such as Bcl-2, COX-2, cyclin D1, VEGF, MM9, and ICAM-1. In summary, through its antioxidant and anti-tumor effects, curcumin sensitizes the cells to radiotherapy (92).

Yang et al. found that in breast cancer stem cells, curcumin bound to glucose-conjugated gold nanoparticles significantly reverses radioresistance under hypoxic conditions. The molecular mechanism involves inhibiting hypoxia-inducible factor 1-alpha (HIF-1α) and heat shock protein 90 (HSP90) expression while increasing ROS levels, confirming the radiosensitizing effect of curcumin on breast cancer (93).

The efficacy of combining curcumin with radiotherapy has been preliminarily explored in small-sample clinical studies. A randomized, double-blind, placebo-controlled clinical trial involving 30 breast cancer patients undergoing radiotherapy found that co-administration of curcumin during radiotherapy reduced the severity of radiation-induced dermatitis (94). Similarly, another randomized, triple-blind, placebo-controlled trial on ‘The Impact of Nanocurcumin on Radiation-Induced Skin Reactions in Breast Cancer Patients’ indicated that although the effect was not significant, the use of nanocurcumin alleviated radiation-induced skin toxicity, particularly in pain relief (154).

### 4.2 Scutellaria baicalensis

Scutellaria baicalensis, commonly known as Huangqin, is a traditional Chinese medicinal herb, and its roots are extensively used in traditional Chinese medicine. Huangqin contains various chemical components, among which flavonoids, including Wogonin, baicalein, baicalin, are some of the major constituents. These components are believed to possess multiple pharmacological activities, including anti-inflammatory, antioxidant, antimicrobial, and anti-tumor effects. Wogonin (5,7-dihydroxy-8-methoxyflavone) is an O-methylated flavone, belonging to the polyphenolic flavonoid class. This compound is derived from the roots of Huangqin or traditional Chinese medicine Huangqin and has been used in traditional Chinese medicine to treat hepatitis, diarrhea, infections, inflammation, hypertension, cardiovascular diseases, neurodegenerative diseases, and tumors.

Scutellaria baicalensis and its active components (baicalein, baicalin, wogonin, wogonoside, oroxylin A, and skullcapflavone) exhibit anti-breast cancer activity through various mechanisms, including inhibiting proliferation, inducing apoptosis, blocking invasion and metastasis, overcoming drug resistance, and regulating non-coding RNA. Additionally, mechanisms such as senescence, autophagy, angiogenesis, and glycolysis play roles in their anti-breast cancer activity. Moreover, multiple signaling pathways contribute to the anti-tumor effects of Huangqin, such as NF-κB, Wnt/β-catenin, SATB1, Bcl-2 family proteins, Caspase, PI3K/Akt, mTOR, ERK, p38-MAPK, TGF-β/Smad, and Hippo/YAP pathways (95).

Research by Park J R and colleagues found that Huangqin extract (SBGE) acts on MCF-7 breast cancer cells, increasing the sub-G1 phase ratio, inhibiting mitochondrial membrane potential, and inducing cell apoptosis by downregulating Bcl-2 and upregulating Bax. Huangqin induces caspase-dependent apoptosis in MCF-7 breast cancer cells by activating the mitogen-activated protein kinase (MAPK) signaling pathway and inducing reactive oxygen species generation (96). Studies by CZ Wang et al. revealed that a Huangqin extract (SbF1) without baicalin blocks the S and G2/M phases of human breast cancer MCF-7 cells, significantly increasing apoptosis induction (97). Wogonin inhibits stem cell-like features (such as mammosphere formation, side population, Oct3/4 and ABCG2 expression, and CD44highCD24low population) and induces apoptosis in radiation-resistant and chemoresistant MDA-MB-231 human breast cancer cells by upregulating IFIT2 (98).

Several studies have identified Nrf2 and HIF-1α signaling pathways as important targets for breast cancer radioresistance (99). Wang et al. identified upregulation of Nrf2 and HIF-1α in radioresistant breast cancer cells. They demonstrated that the combination of Wogonin and radiation therapy effectively suppressed DNA methylation and histone deacetylation in radioresistant breast cancer cells, leading to epigenetic upregulation of CpG site methylation in the Keap1 promoter region. Subsequently, this epigenetic modification resulted in decreased expression of Nrf2 and HIF-1α, significantly promoting apoptosis in breast cancer cells and reducing radiation resistance in breast cancer (100). However, some research indicates that Wogonin treatment reduces the survival rate of MCF-7 breast cancer cells in a dose- and time-dependent manner. Pre-treatment of cells with 5 and 10 µM concentrations of Wogonin for three days before irradiation resulted in increased radioresistance compared to untreated cells, suggesting that Wogonin induces radioresistance in MCF-7 breast cancer cells (155). Therefore, further in-depth research is needed to explore the effective components and doses of Huangqin in overcoming radioresistance.

Furthermore, Huangqin demonstrates the ability to overcome radioresistance in various common malignant tumors. Wogonin regulates the radiation sensitivity of cervical cancer cells *in vitro* through miR-183 and the JAK2/STAT3 signaling pathway (101). Wogonin inhibits the progression of esophageal squamous cell carcinoma by targeting HIF-1A and enhances its sensitivity to radiotherapy (102). Wogonin may reverse radioresistance in colorectal cancer by acting on the SULT2B1 target (103).

### 4.3 Artemisinin

Artemisinin, derived from the Artemisia annua plant, is a natural compound primarily used to treat malaria, fever, and neurological disorders. Additionally, it exhibits anticancer activity, and recent studies suggest its potential as an anti-tumor agent against various solid tumors, including breast cancer.

In human breast cancer cells MDA-MB-435 with p53 mutations, treatment with artemisinin on top of radiotherapy results in higher micronucleus frequency (MNF) and micronucleated cell frequency (MNCF), possibly leading to increased radiosensitivity (104). Polyphenols extracted from artemisinin exhibited anticancer effects in MDA-MB-231 human breast cancer cells through the inhibition of protein expressions associated with cancer stem cells (CSCs), the epithelial to mesenchymal transition (EMT), and cancer progression markers. These markers include overexpressed stem cell markers (CD44 and Oct 3/4), β-catenin, and MMP-9, which are observed in radioresistant breast cancer cells (105). Dihydroartemisinin induces the generation of reactive oxygen species (ROS) and inhibits glutathione-S-transferase (GST) activity, thereby enhancing radiosensitization in human glioma cells (106). The semi-synthetic derivative of artemisinin, artesunate, selectively downregulates survivin, increasing radiosensitivity in neuroblastoma cells by inducing apoptosis, cell cycle arrest, and hindering DNA damage response (107). Artesunate enhances radiosensitivity in human non-small cell lung cancer A549 cells by increasing NO production and inducing G2/M cell cycle arrest (108). Artesunate induces radiosensitivity in cervical cancer cells by causing G2/M cell cycle arrest and apoptosis both *in vitro* and *in vivo (* 109).

### 4.4 Resveratrol

Resveratrol (3,5,4’-trihydroxy-trans-stilbene) is a polyphenol found in common foods such as pistachios, peanuts, mulberries, blueberries, and grapes. Renowned for its diverse biological activities and medicinal properties, resveratrol is one of the most well-known and distinctive stilbene derivatives. It exhibits antioxidant, anti-inflammatory, cardioprotective, neuroprotective, and anticancer effects, making it a therapeutic agent for various diseases, including diabetes, cardiovascular disorders, inflammation, and cancer. Resveratrol demonstrates a dual nature (110); on one hand, it can enhance cellular radioresistance, particularly advantageous for protecting healthy tissues during radiotherapy. On the other hand, numerous studies suggest that it can increase cancer cells’ sensitivity to radiation (111).

In a study, the radiosensitizing mechanism of resveratrol was associated with increased autophagy and apoptosis (112). Mikami et al. also demonstrated that resveratrol could enhance radiation efficacy through the Regenerating Islet-Derived (REG) III expression pathway (113). In other research, combining resveratrol with ionizing radiation delayed the repair of radiation-induced DNA double-strand breaks (DSB) and prolonged G2/M phase arrest, inducing apoptosis (114).

A study found that adding resveratrol treatment to irradiated breast cancer MCF-7 cells induced necrosis/senescence. Furthermore, it observed the activation of the extrinsic apoptosis pathway by reducing the Bax/Bcl-2 ratio, increasing caspase 8 activity, affecting oxidative cell metabolism, elevating oxidative proteins, lipids, and membrane damage, while reducing antioxidant enzyme activity. Resveratrol exhibits radiosensitizing effects on breast cancer (115). Similarly, research confirms that the synergistic action of resveratrol and radiation enhances the expression of apoptosis genes, such as Bax, p53, and caspase 8, leading to cell apoptosis (116). Resveratrol analogue HS-1793 improves tumor tissue perfusion and hypoxia status under low oxygen conditions by inhibiting HIF-1α, suppressing angiogenesis, and enhancing radiosensitivity in mouse breast cancer cells (117). Another study suggests that HS-1793 enhances the efficacy of radiotherapy by inducing antitumor immunity in breast tumor growth (118).

### 4.5 Huaier

Huaier, also known as Poria fungus, is an official fungus used in Traditional Chinese Medicine (TCM). It is a sandy-colored mushroom belonging to the phylum Basidiomycota. It is believed to have various medicinal properties such as clearing heat, detoxification, nourishing blood, and moistening yin. In cancer treatment, extracts from Huai’er have demonstrated diverse biological functions. Huaier extracts exhibit anti-tumor effects in various malignant tumors (119), with mechanisms including sensitizing to radiotherapy, inducing apoptosis, anti-angiogenesis, reversing drug resistance, inhibiting metastasis, and activating the immune system (120, 121).

According to Ding et al., based on HTA 2.0 microarray results, Huai’er extracts were found to damage genes related to the cell cycle, cell division, cell cycle phases, and DNA repair. Treatment of cells with Huaier at a concentration of 4 mg/ml for 24 hours resulted in a significant increase in the proportion of cells in the G0/G1 phase. Subsequently, within 24 hours after irradiation, MCF-7 cells exposed to 6 Gy of radiation exhibited a G2/M phase block. However, in cells pre-treated with Huaier, this radiation-induced G2/M phase block was not prominently observed. Ku70 and Ku86 are markers of non-homologous end joining (NHEJ), while RAD51 is associated with homologous recombination (HR). In both MCF-7 and MDA-MB-468 cells, Huaier pre-treatment led to a time-dependent decrease in RAD51 levels following irradiation with 6 Gy (at 0, 2, 6, and 24 hours) compared to control cells. However, there were no significant changes in the protein levels of Ku70 and Ku86. Subsequent *in vitro* experiments confirmed that Huaier, by downregulating proteins associated with cell cycle regulation in MCF-7 and MDA-MB-468 breast cancer cells, induced G0/G1 arrest, prolonged the duration of γ-H2Ax foci after radiotherapy, and increased the radiosensitivity of breast cancer cells by downregulating RAD51, disrupting the homologous recombination (HR) pathway for DNA repair (122).

### 4.6 Berberine

Berberine, an isoquinoline alkaloid derived from various medicinal herbs such as Coptis chinensis, exhibits a wide range of pharmacological and biochemical effects. Known for its antimicrobial, anti-inflammatory, and anti-tumor properties, berberine is extensively used in China to treat gastrointestinal discomfort. Furthermore, berberine demonstrates anti-tumor activity against various cancer cells, often through the inhibition of cell cycle progression and promotion of apoptosis (123–129).

Treating MCF-7 and MDA-MB-468 breast cancer cells with berberine at a concentration of 15 μM and subjecting them to various doses of X-rays (ranging from 1 to 4 Gy), resulted in a decrease in RAD51 protein levels compared to control cells. Specifically, in cells pre-treated with 15 μM berberine for 24 hours, the levels of RAD51 protein were significantly reduced at specific time points after X-ray irradiation (0, 2, 6, and 24 hours). These findings highlight the potential of berberine as a radiosensitizer in the treatment of human breast cancer, as it exerts its effects through G2/M phase cell cycle arrest and the downregulation of the homologous recombination repair protein RAD51, ultimately increasing the therapeutic efficacy of radiation therapy (130). Monitoring changes in the expression of the tumor stem cell markers OCT4 and SOX2 reveals that berberine enhances the cytotoxic effect of radiotherapy by targeting cancer stem cells (131). Additionally, 13-ethyl berberine (13-EBR) exerts pro-apoptotic effects in radiotherapy-resistant breast cancer cell lines by inducing mitochondrial ROS production and activating the mitochondrial apoptosis pathway (132).

In a study by Zeng et al., low-dose berberine modulates the tumor microenvironment through the PI3K/HIF-1 pathway, overcoming radiation resistance in cervical cancer cells under low glucose and hypoxic conditions (133). Wang et al. discovered that berberine enhances the radiosensitivity of osteosarcoma by targeting Rad51 and inhibiting epithelial-mesenchymal transition (134). Liu et al. found that berberine increases the radiosensitivity of esophageal cancer cells by downregulating the homologous recombination repair protein RAD51 (135).

### 4.7 Triptolide and celastrol

Triptolide and Celastrol are two natural compounds extracted from the Tripterygium wilfordii. In traditional Chinese medicine, they have been used to treat inflammatory conditions such as rheumatoid arthritis, and in recent years, they have garnered scientific attention due to their diverse biological activities, including anti-inflammatory, antioxidant, and anti-tumor effects.

Triptolide, a diterpenoid triepoxide extracted from the Thunder God Vine in China, exhibits the ability to inhibit cancer cell proliferation *in vitro* and suppress tumor growth and metastasis *in vivo*. Jiang et al. reported that triptolide inhibits the growth of breast cancer cells by reducing HMGB1 expression both *in vitro* and *in vivo (* 136). Triptolide induces autophagy and affects the phosphorylation of p38 mitogen-activated protein kinase, extracellular signal-regulated kinase (Erk) 1/2, and mammalian target of rapamycin (mTOR), leading to autophagy and apoptosis in breast cancer cells (137).

In a study by Li et al., triptolide significantly inhibited the expression of GRP78 protein in radiotherapy-resistant nasopharyngeal carcinoma cells (CNE2-SR) and induced apoptosis and M/G2 phase arrest, suggesting that triptolide may serve as a potential radiosensitizer for NPC treatment (138). Triptolide markedly increased the radiosensitivity of human glioma U251 cells by suppressing the PI3K/Akt signaling pathway (139). In comparison to radiation alone, lung cancer cells treated with triptolide in combination with radiation promoted apoptosis, with observed increases in PARP cleavage, JNK, and p53 expression, while HSP70 and Akt expression were inhibited (140).

Celastrol, a triterpenoid compound extracted from the Thunder God Vine, is commonly used to treat inflammation and autoimmune diseases. Recently, the potential anti-cancer activity of celastrol has gained widespread attention. Celastrol can inhibit the proliferation of various tumor cells and suppress tumor initiation, progression, and metastasis in various cancer models. Celastrol regulates the expression of pro-inflammatory cytokines, MHC II, HO-1, iNOS, NF-κB, Notch-1, AKT/mTOR, CXCR4, TRAIL receptors DR4 and DR5, CHOP, JNK, VEGF, adhesion molecules, proteasome activity, topoisomerase II, potassium channels, and heat shock response (141).

Gao et al. found that celastrol enhances TRAIL-induced apoptosis by downregulating cell survival proteins, including cFLIP, IAP-1, Bcl-2, Bcl-xL, survivin, and XIAP, and upregulating death receptor expression through ROS-mediated CHOP pathway (142). Studies on a mouse lung cancer model revealed that the novel radiosensitizer celastrol, when combined with ionizing radiation, has therapeutic effects, maximizing the treatment efficacy against non-small cell lung cancer (143). In non-small cell lung cancer cells, celastrol mediates radiation sensitization through the modification of the antioxidant molecule thioredoxin. Research indicates that celastrol impairs DNA damage processing and increases apoptosis in prostate cancer PC-3 cells, rendering PC-3 cells sensitive to radiation both *in vitro* and *in vivo (* 144).

### 4.8 Withaferin

Withaferin is a steroidal compound found in Ashwagandha, also known as Indian ginseng, and is believed to possess various pharmacological activities, including potential anticancer effects, anti-inflammatory properties, and neuroprotective effects.

U937 lymphoma cells were treated with different concentrations of withaferin A (0, 0.3, 0.5, and 1 μM) along with increasing doses of X-ray radiation (from 0 to 10 Gy). However, most experiments were conducted under conditions of sublethal doses of 0.5 μM withaferin A and 10 Gy radiation, as under these conditions, withaferin A in combination with radiation effectively induced nearly 40% of cell death, accompanied by typical morphological changes indicative of apoptosis, such as cell shrinkage, cytoplasmic condensation, and nuclear condensation. Administration of withaferin A followed by radiation resulted in increased levels of ROS production, upregulation of PARP expression, downregulation of Bcl-2, and activation of JNK and p38 signaling pathways. These pathways are known to be activated in response to various cellular stressors, such as ROS (145). Similar effects were observed in other cell lines within the same study, including Caki (renal cancer), SK-Hep1 (hepatic cancer), MDA-MB-231 (breast cancer), and HeLa (cervical cancer) cells, when treated with 4 μM of Withaferin A followed by exposure to 10 Gy of X-ray radiation (146).

In *in vitro* experiments with breast cancer cells, Withania induced G2/M and pre-G1 phase arrest in MDA-MB-231 cells and pre-G1 phase arrest in MCF7 cells. Flow cytometry analysis revealed that in the experimental group, consisting of MDA-MB-231 and MCF7 breast cancer cell lines treated with Withania prior to irradiation, there was an increased percentage of apoptotic cells (including early, late, and necrotic cells) compared to the control group, which only received irradiation. Additionally, it altered BCL2/Bax signal transduction and triggered apoptosis in breast cancer cells exposed to γ radiation (147).

### 4.9 Caffeic acid phenethyl ester

Caffeic acid phenethyl ester (CAPE) is a major polyphenol extracted from bee propolis. CAPE exhibits various medicinal properties, including antiviral, anti-inflammatory, and antioxidant effects. It is considered to have anticancer effects on different tumor cell lines and has been confirmed as a radiosensitizer in certain types of cancer.

CAPE enhances the radiosensitivity of MDA-MB-231 (estrogen receptor-negative) and T47D (estrogen receptor-positive) breast cancer cell lines by prolonging radiation-induced DNA damage. CAPE inhibits clonogenic formation and sustains radiation-induced DNA damage in both cell lines, with a more pronounced effect in T47D cells. Given the structural similarity between CAPE and estrogen, CAPE may be more effective in estrogen receptor-positive T47D cells than in estrogen receptor-negative MDA-MB-231 cells (148). Pre-treating MDA-MB-231 and T47D breast cancer cells with 1 µM CAPE for 72 hours prior to irradiation with 6 Gy and 8 Gy, the experiment measured the quantity of DNA strand breaks at four different time points. The results demonstrated that CAPE reduced the viability of both cell lines in a dose- and time-dependent manner. The radiosensitizing ability of CAPE in breast cancer cells may primarily operate through DNA damage repair mechanisms. Combination treatment of CAPE with gamma-ray therapy renders androgen-independent prostate cancer DU145 and PC3 cells more sensitive to radiation. Mechanistically, combined treatment induces γ H2AX foci and apoptosis, enhances Akt/mTOR phosphorylation, and impedes cell migration. The joint action of CAPE and ionizing radiation (IR) leads to intensified DNA damage and cell death by reducing RAD50 and RAD51 proteins involved in DNA repair (149). Furthermore, CAPE treatment sensitizes lung cancer cells to radiation by promoting apoptosis and inducing cell cycle arrest at S/G2 phase, associated with post-treatment depletion of glutathione (150). In mice hepatoma (H22) cells, co-treatment with CAPE and 60Coγ radiation shows potential radiosensitizing effects, as evidenced by reduced cell viability, altered cell cycle, increased apoptosis, and elevated reactive oxygen species (ROS) levels. Additionally, high-throughput sequencing identified 46 differentially expressed lncRNAs in H22 cells post-CAPE treatment, including 24 upregulated and 22 downregulated lncRNAs. Representative lncRNAs (LNC-004553, LNC-000751, and LNC-000561) were selected for validation using quantitative real-time polymerase chain reaction (qRT-PCR) after Gene Ontology (GO) and Kyoto Encyclopedia of Genes and Genomes (KEGG) analyses (151). CAPE treatment sensitized CT26 colorectal adenocarcinoma cells to radiation, possibly through GSH depletion and inhibition of NF-κB activity, without causing toxicity to bone marrow, liver, and kidneys (152). CAPE exerts antiproliferative and radiosensitizing effects on medulloblastoma cells by reducing the G2/M fraction, downregulating cell cycle protein B1 expression, and promoting apoptosis (152). Subsequent studies support the anti-proliferative and radiosensitizing effects of CAPE on medulloblastoma, likely achieved through GSH consumption, increased ROS activity, and NF-κB activity inhibition (153).

## 5 Future directions

Breast cancer is the most common malignancy in women and ranks second in female malignancy-related mortality. Radiotherapy plays a crucial role in the comprehensive treatment of breast cancer, with different molecular subtypes corresponding to distinct treatment strategies and prognoses. Considering cancer characteristics, overcoming radiotherapy resistance to enhance tumor cure rates is a pressing issue for scientists. A systematic elucidation of the molecular mechanisms underlying radiotherapy resistance in breast cancer can assist in the targeted selection of appropriate drugs to improve radiotherapy sensitivity.

Traditional Chinese medicine (TCM) has a rich history spanning thousands of years, offering abundant resources for further drug development. This review highlights potential herbal candidates to enhance radiotherapy sensitivity in breast cancer. Natural compounds derived from traditional Chinese herbs, such as curcumin, baicalein, artemisinin, resveratrol, Tremella fuciformis, berberine, triptolide, celastrol, withaferin, and caffeic acid phenethyl ester, have shown efficacy in increasing radiotherapy sensitivity in breast cancer. Curcumin, through its ability to promote intrinsic and extrinsic apoptosis and inhibit the expression of proteins associated with cell survival, proliferation, angiogenesis, invasion, and metastasis, exerts its antioxidant and anti-tumor effects, rendering cells sensitive to radiation therapy. Wogonin can epigenetically regulate the Keap1 gene, inhibiting the Nrf2/HIF-1α pathway, inducing apoptosis in breast cancer cells, and alleviating acquired radioresistance. Polyphenols derived from artemisinin exhibit anticancer effects in radiotherapy-resistant MDA-MB-231 human breast cancer cells by inhibiting the protein expressions associated with CSCs, EMT, and cancer progression markers. The radiosensitizing mechanism of resveratrol is associated with increased autophagy and cell apoptosis. Huaier downregulates proteins associated with cell cycle regulation in breast cancer cells, thereby regulating G0/G1 cell cycle arrest. It also downregulates RAD51, which interferes with the homologous recombination (HR) pathway involved in DNA damage repair, resulting in increased radiosensitivity of breast cancer cells. Berberine enhances radiation sensitivity in irradiated breast cancer cells by regulating cell cycle arrest at the G2/M phase and downregulating RAD51 protein. Withaferin, in combination with radiation therapy, induces G2/M and pre-G1 phase arrest in breast cancer cell lines. It modulates the BCL/BAX protein family to promote cell apoptosis, thereby increasing the radiosensitivity of breast cancer cells. Caffeic acid phenethyl ester (CAPE) reduces DNA damage repair capacity in breast cancer cells, which is beneficial in overcoming resistance to radiation therapy.

However, due to the complex composition of traditional Chinese medicine, it may contain hundreds of different chemical components. These components can interact in multiple ways, thereby producing sensitizing effects in radiotherapy. Currently, our understanding of the specific mechanisms by which herbal medicine components enhance radiotherapy remains limited, requiring further research to unravel their mysteries. At present, research on the relationship between traditional Chinese medicine and resistance to radiotherapy in breast cancer is mostly based on *in vitro* experiments. There is a lack of large-sample clinical trial evidence to support the radiosensitizing effect of traditional Chinese medicine on breast cancer. Additionally, the sensitizing effects of TCM in radiotherapy may vary among individuals, and determining the appropriate dosage of TCM is also a significant concern. In the future, a broader range of research and clinical trials should be conducted to attain a comprehensive understanding of this field.

## Data availability statement

The original contributions presented in the study are included in the article/supplementary material. Further inquiries can be directed to the corresponding authors.

## Author contributions

XZ: Writing – original draft. TL: Writing – original draft. YQ: Writing – original draft. ZY: Writing – review & editing. DW: Writing – review & editing. ZW: Writing – original draft, Writing – review & editing. JZ: Writing – original draft, Writing – review & editing. ZB: Writing – original draft, Writing – review & editing.
